# Editorial: Intervertebral disc degeneration: mechanisms and therapeutics

**DOI:** 10.3389/fcell.2024.1401933

**Published:** 2024-04-18

**Authors:** Futong Wu, Xiaolong Chen, Sidong Yang, Xiao Lv, Felicity Han, Hongfei Xiang

**Affiliations:** ^1^ The Affiliated Hospital of Qingdao University, Qingdao, China; ^2^ University of New South Wales, Kensington, NSW, Australia; ^3^ The University of Queensland, Brisbane, QLD, Australia; ^4^ Huazhong University of Science and Technology, Wuhan, Hubei, China

**Keywords:** intervertebral disc degeneration (IDD), low back pain, sciatic, mechanism, therapeutic

Low back pain (LBP) is a prevalent musculoskeletal disorder, affecting approximately 70% of individuals at some point in their lives (Hoy et al., 2010). Sciatica, characterized by leg pain associated with LBP, represents one of the most common manifestations of lower back pain. It is estimated that around 5% of men and 2.5% of women will experience sciatica during their lifetime (Konstantinou and Dunn, 2008). Intervertebral disc degeneration (IVDD) stands out as the predominant underlying cause for both LBP and sciatica occurrences. IVDD may be linked to a range of intricate lifestyle-related factors, including age, gender, depression, social isolation, educational background, alcohol consumption, smoking habits, obesity, and physical activity (PA) (Mikkelsson et al., 2006; Shiri et al., 2013). Henceforth, it is imperative to delve into the mechanisms underlying these spinal disorders to prevent their occurrence and devise more efficacious treatment approaches. This Research Topic encompasses an assemblage of original research and review articles aiming to shed light on the latest advancements pertaining to the underlying mechanisms of IVDD and the development of therapeutic strategies targeting them.

PA, which involves energy-consuming musculoskeletal movements, is closely associated with IVDD. While numerous observational studies suggest that PA may potentially protect against LBP, traditional observational studies have several limitations, including confounding factors and reverse causation (Mok et al., 2019; Lemes Í et al., 2022). Therefore, further investigation is required to explore the causal relationships between PA and LBP. In this Research Topic, Guan et al. conducted an original research article to investigate the causal relationship between PA and three spinal disorders: LBP, IVDD, and sciatica. They employed a two-sample Mendelian randomization strategy using openly accessible data from genetic instruments derived from genome-wide association studies. By utilizing genetic variation as an instrumental variable, the study aimed to assess the causal link between PA and these spinal disorders. Additionally, sensitivity analyses were performed to evaluate the robustness of the analysis results in case of any violation of Mendelian randomization assumptions. This study offers a more precise evaluation of the impact of PA on LBP, IVDD, and sciatica. Previous studies have identified some correlation between PA and LBP, IVDD, and sciatica; however, the consistency in the effect of different methods and intensities used to assess PA on this association remains inconclusive. Furthermore, the causal relationship between PA and IVDD, as well as sciatica, is still uncertain. Although our findings suggest that there is no causality between various assessment methods and intensity levels of PA with IVDD and sciatica, it does not dismiss the existence of an association between PA and intervertebral discs. This research methodology demonstrates high internal validity and reliability while providing a crucial foundation for preventing and treating these spinal conditions.

IVDD is a multifaceted and ongoing process, intricately associated with the dysregulation in the synthesis and breakdown of extracellular matrix (ECM), triggered by various factors including progressive aseptic inflammatory response and age-related reduction in nucleus pulposus cells (NPCs). This intricate process may involve the uncoordinated action of a variety of regulatory molecules such as cytokines, growth factors, and degradative enzymes. The presence of a suboptimal ECM environment resulting from chronic inflammation can lead to increased susceptibility of drugs and other bioactive preparations to degradation. Hydrogels, serving as scaffolds and carriers for cells/drugs, possess advantageous properties such as resistance to degradation and sustained release of loaded drugs due to their high hydration characteristics. Consequently, hydrogels play a crucial role in the treatment of nucleus pulposus (NP) degeneration and various aspects related to IVDD (Ma et al., 2019). In this Research Topic, a review article by Lui et al. provides a concise overview of the anatomy, pathology, and current treatment approaches for IVDD. The article also presents a comprehensive analysis comparing the advantages and disadvantages of various hydrogel materials, along with potential modifications to overcome existing limitations. These hydrogels exhibit promising potential as cell carriers for intervertebral disc tissue repair while simultaneously modulating immune responses and facilitating tissue regeneration. The authors also specifically introduce “smart hydrogels” - stimuli-responsive hydrogels, which have promising potential applications in the treatment of IVDD. These include inflammation-responsive hydrogels, temperature-sensitive hydrogels, enzyme-reactive hydrogels, light-sensitive hydrogels, and other stimulus-responsive hydrogels. This type of hydrogel exhibits a sol state upon entering the lesion site. It can rapidly form a gel or release loaded drugs under specific conditions such as light exposure, temperature changes, pH variations, etc. It offers long-lasting anti-inflammatory effects and on-demand intelligent drug release capabilities while providing sufficient mechanical support. Due to its unique advantages, this technology has gained significant attention in the field. Similarly, various bioactive compounds possess the capability to effectively penetrate the NP by delivering biomaterials, thereby facilitating the replenishment of damaged NP tissues, alleviating the inflammatory microenvironment within NP, rectifying disrupted ECM metabolism, and ultimately restoring the structural and mechanical properties of intervertebral discs (Wu et al., 2022). Song et al. have published a concise review article on bioactive substances that possess the potential to delay and reverse the progression of IVDD. These substances encompass various therapeutic modalities such as cellular therapy, genetic therapy, small molecule drugs, other agents, and combination drug delivery systems. Additionally, diverse biological material delivery systems with distinct biological properties and release characteristics, including nano micelles, microspheres, and hydrogels, are explored. By incorporating these therapeutic factors into specific local delivery systems, the study aims to achieve precise treatment of IVDD by targeting metabolic changes, alterations in the microenvironment, and even gene pathways associated with IVDD. The ultimate goal is to halt or potentially reverse the degenerative process of IVDD. Furthermore, various fundamental biomaterials can be utilized to construct these innovative delivery systems. By utilizing diverse molecular and chemical modifications, a plethora of biomaterials with distinct properties can be acquired to fulfill various requirements. In conclusion, this research proposes a methodology for the precise treatment of IVDD by introducing therapeutic factors and biomaterials as delivery tools, thereby addressing the Research Topic of LBP management.

Regenerative therapy aimed at restoring the physiological structure and biomechanical function of intervertebral discs has garnered significant attention in recent years, with a particular focus on repairing disc tissue to slow or reverse the decline of ECM and NPCs. Among these approaches, mesenchymal stem cells (MSCs) have emerged as the most extensively studied for treating IVDD. MSCs, being pluripotent stem cells capable of differentiating into osteoblasts, chondrocytes, and adipocytes, have emerged as the preferred material for intervertebral disc regeneration due to their abundant sources, easy accessibility, minimal immunogenicity, differentiation-inducing solid ability, and robust proliferation even in low oxygen and glucose environments (Pittenger et al., 1999; Miao et al., 2006). While bone marrow mesenchymal stem cells (BMSCs) were previously considered the gold standard for regenerative therapy, recent studies have demonstrated that umbilical cord mesenchymal stem cells (UCMSCs) surpass BMSCs in terms of cell origin and differentiation potential. In this Research Topic, Huang et al. published a comprehensive review article elucidating the utilization of UCMSCs for regenerative therapy targeting IVDD. The article highlights the remarkable proliferative and differentiating potential of UCMSCs, enabling their differentiation into NPCs, facilitation of ECM synthesis, and regulation of inflammatory responses within the intervertebral disc microenvironment. Augmenting the therapeutic efficacy of UCMSCs can be achieved through their integration with biomaterial scaffolds exhibiting exceptional performance characteristics. Notably, overcoming challenges associated with harsh intervertebral disc conditions and mechanical loading is crucial in maximizing the regenerative capacity of UCMSCs. Consequently, harnessing their regenerative effects can be enhanced by attaching UCMSCs to biomaterial scaffolds possessing superior properties - an innovative approach worth exploring further. In addition, the paracrine action of MSCs has been demonstrated to facilitate the acquisition of exosomes with lipid bilayers, which have shown potential in promoting tissue repair and regeneration. This finding offers a novel perspective for further investigation into UCMSCs. The Huang et al. study also acknowledges the limitations associated with UCMSCs and proposes potential solutions and future research directions. However, it also highlights the scarcity of relevant clinical trials, emphasizing the necessity for additional studies to validate their efficacy and safety. Consequently, this article serves as a valuable reference for advancing research and application of UCMSCs while providing innovative ideas and methodologies for the regenerative treatment of IVDD. Additionally, dysregulation of BMSCs occurs specifically in Modic type 1 change (MC1), leading to an increased number of BMSCs in the perivascular bone region (Shi et al., 2019). BMSCs play a role in blood vessel and nerve formation/remodeling within the bone marrow microenvironment, with various MSCs exhibiting pro-neurotrophic effects (Brohlin et al., 2012; Heggli et al., 2021). However, it remains unclear whether dysregulated MC1 BMSCs actively promote nerve growth. In an original research article, Mengis et al. postulated that dysregulated MC1 BMSCs significantly facilitated the augmentation of MC1 bone marrow innervation. They elucidated the regulatory mechanism of BMSCs on neurite outgrowth by co-cultivating MC1 BMSCs with neuronal model cells (SH-SY5), a neuroblastoma cell line, and conducting transcriptome analysis and gene set enrichment analysis. The findings demonstrated significant enrichment of MC1 BMSCs in the gene set associated with the brain-derived neurotrophic factor (BDNF) signaling pathway, particularly in relation to the BDNF TRKB signaling pathway, when compared to the control group. Furthermore, the regulatory mechanisms of MC1 neurite growth are addressed by BMSCs through modulation of gamma-aminobutyric acid (GABA) neurotransmitter conduction and ECM degradation. This investigation elucidates that BMSCs promote neurite growth via regulation of BDNF signaling pathway and GABA neurotransmitters, providing a theoretical foundation for future research on MC1 treatment aimed at blocking the neurotrophic activity of BMSCs to improve patients’ symptoms.

In conclusion, this research theme provides a comprehensive overview of the diverse mechanisms contributing to IVDD and their corresponding treatment options. It is anticipated that this investigation will foster increased interest in unraveling the underlying pathophysiological processes involved in disc degeneration and exploring potential therapeutic strategies for its management.
